# Validation of Consumer-Grade 3D Scanners for Clinical Facial Reconstruction: Comparison with a Professional Structured-Light Reference System

**DOI:** 10.3390/bioengineering13080941

**Published:** 2026-08-20

**Authors:** Bibiána Ondrejová, Branko Štefanovič, Katarína Dudová, Lyzette Yeboah-Kyeremeh, Jaroslav Majerník, Jozef Živčák

**Affiliations:** 1 Department of Biomedical Engineering and Measurement, Faculty of Mechanical Engineering, Technical University of Košice, Lerná 1/9, 040 01 Košice, Slovakia; bibiana.ondrejova@tuke.sk (B.O.); branko.stefanovic@tuke.sk (B.Š.); jozef.zivcak@tuke.sk (J.Ž.); 2 Department of Medical Informatics and Simulator Medicine, Faculty of Medicine, Pavol Jozef Šafárik University in Košice, 040 11 Košice, Slovakia; jaroslav.majernik@upjs.sk

**Keywords:** 3D scanning, facial anthropometry, reconstructive surgery, burn medicine, structured-light scanning, geometric accuracy

## Abstract

Three-dimensional facial scanning is an important tool in reconstructive surgery and burn medicine for objective documentation, treatment planning, and fabrication of patient-specific devices. While professional structured-light scanners provide high geometric accuracy, their cost limits routine implementation. Several low-cost consumer-grade alternatives are available; however, their accuracy for clinically relevant facial applications remains insufficiently validated. Thirty volunteers were initially recruited for facial scanning. Two participants withdrew from further scanning for personal reasons, resulting in a main study cohort of 28 participants. Complete datasets from all 28 participants were available for Artec Eva, Revopoint MIRACO, and Creality CR-Scan 01, whereas 22 Kiri Engine datasets met the predefined quality criteria for smartphone photogrammetry analysis. Facial scans acquired using Revopoint MIRACO, Creality CR-Scan 01, and Kiri Engine were compared with a professional structured-light scanner (Artec Eva) used as the reference system. Six clinically relevant inter-landmark distances were analysed together with global surface deviation relative to Artec Eva reference using Cloud-to-Mesh analysis. Agreement was evaluated using mean signed differences, mean absolute error (MAE), intraclass correlation coefficients (ICC), Bland–Altman analysis, and post-hoc statistical testing. Both MIRACO and CR-Scan demonstrated favourable geometric agreement with the Artec Eva reference. Global surface RMSE relative to Artec Eva reference was 0.72 ± 0.24 mm for MIRACO and 0.57 ± 0.17 mm for CR-Scan, compared with 1.41 ± 0.58 mm for Kiri Engine. More than 90% of facial surface points were within 1 mm of the reference model for MIRACO (91 ± 8%) and CR-Scan (93 ± 7%), whereas Kiri Engine achieved 68 ± 16%. Pooled ICC values ranged from 0.998 to 0.999 across the evaluated systems. Pairwise comparisons showed no significant difference between MIRACO and CR-Scan (*p* = 0.42), while both significantly outperformed Kiri Engine (*p* < 0.001). Consumer-grade structured-light scanners demonstrated submillimeter global surface deviations, supporting further investigation for facial surface documentation and monitoring applications. Smartphone photogrammetry showed substantially larger deviations and may be less suitable for applications requiring precise quantitative measurements. Low-cost structured-light systems demonstrated promising geometric performance in healthy volunteers; however, further validation in relevant clinical populations is required before their applicability to reconstructive surgery and burn care workflows can be established.

## 1. Introduction

Three-dimensional (3D) surface imaging has become increasingly important in reconstructive surgery and burn medicine. Accurate digital representations of facial anatomy enable objective documentation of deformities, longitudinal monitoring of treatment outcomes, surgical planning, and fabrication of patient-specific devices such as compression masks, facial splints, orthoses, and prosthetic components [1,2,3]. In addition, accurate facial surface models constitute an essential component of computer-assisted surgical planning, digital patient documentation, and image-guided reconstructive workflows, where reliable three-dimensional data are required for objective clinical decision-making and treatment evaluation.

Traditional clinical documentation relies predominantly on two-dimensional photography and manual anthropometric measurements. Although widely used, these methods provide limited information regarding facial morphology and may fail to capture subtle three-dimensional or volumetric changes. Three-dimensional surface scanning overcomes many of these limitations by providing comprehensive geometric information in a non-invasive and radiation-free manner [1,4,5].

Professional facial scanning systems, including structured-light scanners and stereophotogrammetric systems, have demonstrated high accuracy and reliability for clinical facial surface acquisition. These systems are frequently used for craniofacial anthropometry, treatment outcome assessment, orthognathic surgery planning, and soft-tissue facial analysis [4,5,6,7,8]. However, their acquisition and maintenance costs, equipment size, and dedicated workflow requirements may limit adoption in smaller hospitals, burn centres, and outpatient facilities [5,9].

Several studies have reported clinically acceptable accuracy and reproducibility of stereophotogrammetric and structured-light facial imaging systems, with many reported deviations around or below 1 mm depending on the device, facial region, reference method, and acquisition protocol [6,7,8,10]. Consequently, these technologies are increasingly used in digital clinical workflows for craniofacial assessment, surgical planning, treatment monitoring, and fabrication of patient-specific medical devices [1,3,4,9]. Despite their demonstrated performance, widespread clinical adoption remains limited by equipment costs, dedicated infrastructure requirements, and restricted portability. As a result, there is growing interest in determining whether low-cost scanning technologies can provide sufficient accuracy for quantitative clinical applications [5,9,10,11,12].

Recent advances in consumer-grade 3D scanning technologies have resulted in the emergence of affordable structured-light scanners, RGB-D sensors, smartphone-based scanning applications, and photogrammetric solutions. These systems offer substantially lower costs and greater accessibility, potentially enabling broader implementation of 3D facial assessment in routine clinical practice [9,11,12]. Nevertheless, evidence regarding their geometric accuracy and suitability for reconstructive and burn-care applications remains limited, particularly when quantitative surface deviation analysis is required.

The aim of this study was therefore to evaluate the geometric agreement of three low-cost facial scanning systems—Revopoint MIRACO, Creality CR-Scan, and Kiri Engine—using the professional structured-light scanner Artec Eva as a reference. Both landmark-based anthropometric measurements and global surface deviation analyses were performed to inform their potential suitability for quantitative facial assessment and for integration into computer-assisted reconstructive surgery, digital treatment planning, and image-guided clinical workflows.

## 2. Materials and Methods

### 2.1. Study Design and Participants

This study was designed as an in vivo comparative validation study evaluating the geometric agreement of three low-cost facial 3D scanning systems against a professional structured-light reference scanner.

Thirty healthy adult volunteers were initially recruited for the study. Two participants withdrew from further scanning for personal reasons, resulting in a main study cohort of 28 participants with complete datasets suitable for the primary comparative analysis. Complete datasets from all 28 participants were available for Artec Eva, Revopoint MIRACO, and Creality CR-Scan 01. In contrast, only 22 of the 28 Kiri Engine datasets met the predefined quality criteria and were therefore included in the corresponding analyses. Six Kiri Engine datasets were excluded due to reconstruction failure, incomplete facial coverage, failed mesh generation, or severe geometric artefacts. The analysed cohort had a mean age of 23.7 ± 4.1 years (range 19–43 years). Twenty participants (71%) were female and eight (29%) were male. Fitzpatrick skin phototypes were distributed as follows: type II (*n* = 10), type III (*n* = 14), and type IV (*n* = 4). Participants with active facial skin disease, significant facial deformity, extensive facial hair interfering with scanning, or inability to maintain a neutral facial expression during acquisition were excluded.

The study was conducted in accordance with the Declaration of Helsinki and approved by the Ethics Committee of AGEL Hospital Košice-Šaca (Slovakia) (protocol code 17-2023 and 16 November 2023).

### 2.2. Scanning Systems

Four facial scanning systems were included in the study, of which Artec Eva served as the reference scanner and the remaining three systems were evaluated against it.

The Artec Eva (Artec 3D, Senningerberg, Luxembourg) was used as the reference scanner for all comparisons in the present study. It is a professional handheld structured-light scanner with a manufacturer-specified three-dimensional accuracy of up to 0.1 mm. Professional optical and structured-light scanning systems are widely used for craniofacial, dental, maxillofacial, and facial prosthetic surface acquisition and have also been employed for the evaluation of portable and low-cost three-dimensional scanning technologies [13,14,15,16]. Artec Eva was selected as the reference system because of its professional-grade structured-light technology, high nominal geometric accuracy, established use in biomedical surface acquisition, non-contact acquisition principle, and suitability for in vivo scanning of human facial surfaces. Its ability to generate high-resolution three-dimensional surface models makes it an appropriate comparative reference for the evaluation of lower-cost scanning systems. However, Artec Eva was used as a comparative reference and was not considered an error-free representation of the true facial geometry. Therefore, all geometric deviation metrics reported in this study should be interpreted as differences relative to the Artec Eva reference surface rather than as absolute measurement errors of the investigated scanning systems.

- The Revopoint MIRACO (Revopoint 3D Technologies, Shenzhen, China) is a portable standalone structured-light scanner equipped with integrated processing hardware and a touchscreen interface. The scanner employs dual infrared structured-light projection and is intended for both close-range and medium-range scanning applications.
- The Creality CR-Scan 01 (Shenzhen Creality 3D Technology Co., Ltd., Shenzhen, China) is a consumer-grade structured-light scanner designed for reverse engineering and digital modelling applications. It combines infrared structured-light projection with RGB image acquisition.
- Kiri Engine (Kiri Innovations Inc., Vancouver, BC, Canada) is a smartphone-based photogrammetry application. In the present study, it was used in photogrammetry mode on an Apple iPhone 16. Reconstruction was performed using cloud-based processing algorithms.

### 2.3. Facial Landmarks and Fiducial Markers

To enable standardized anthropometric measurements, circular adhesive fiducial markers were placed on predefined anatomical landmarks before scanning. The identified landmarks included Trichion (Tr), Glabella (G), Pronasale (Prn), Subnasale (Sn), Gnathion (Gn), Exocanthion (Ex), and Cheilion (Ch). Six clinically relevant inter-landmark distances were subsequently analysed: Trichion–Glabella, Glabella–Gnathion, Glabella–Pronasale, Subnasale–Gnathion, Biocular Width (Ex–Ex), and Mouth Width (Ch–Ch). These measurements were selected because they represent anatomically important dimensions frequently used in reconstructive surgery, craniofacial assessment, and facial outcome evaluation.

### 2.4. Scanning Procedure

All scans were acquired under standardized indoor conditions. Participants were instructed to maintain a neutral facial expression, keep their eyes closed, and avoid head movement throughout acquisition. Hair was secured away from the face whenever necessary to prevent occlusion of anatomical landmarks.

Scanning procedures were performed by a single operator with previous practical experience in three-dimensional surface scanning. Prior to data collection, the operator was familiar with the operation of all scanning systems evaluated in the present study and their respective acquisition procedures. The same operator performed all acquisitions using a standardized scanning protocol to maintain consistency and minimize operator-dependent variability.

Each participant was scanned sequentially using all four systems during a single acquisition session. The scanning sequence was kept consistent for all participants and was as follows: Artec Eva, Creality CR-Scan 01, Revopoint MIRACO, and Kiri Engine. The Artec Eva scan was obtained first and subsequently served as the reference model for all comparisons. Scanning procedures followed manufacturer recommendations regarding acquisition distance, scanning trajectory, and environmental conditions.

For Kiri Engine, photogrammetry mode was used. For each participant, a 15-s video was recorded while the operator moved around the seated subject to obtain overlapping facial views. The video was subsequently uploaded to the Kiri Engine cloud service for automated reconstruction using the highest quality setting available in the application. The resulting textured mesh was exported in OBJ format.

The scanning sequence was not randomized; however, all acquisitions were performed consecutively during the same session under standardized conditions. Therefore, major systematic changes in participant condition between acquisitions were not expected. Nevertheless, a potential effect of the fixed scanning sequence cannot be completely excluded.

### 2.5. Surface Processing and Registration

All three-dimensional models were exported in OBJ format and processed using CloudCompare software v2.13.2 (CloudCompare, Grenoble, France). Prior to analysis, all meshes were cropped to the same facial region, bounded superiorly by the hairline, inferiorly by the lower border of the mandible, and laterally by the pre-auricular skin crease; hair, ears, and the neck were excluded.

An initial point-pair alignment was performed using stable anatomical landmarks, including the glabella, bilateral exocanthions, and pronasale. Landmarks located in more deformable facial regions, particularly around the mouth, were excluded from the initial alignment to minimize potential alignment bias.

Fine registration was subsequently performed using the Iterative Closest Point (ICP) algorithm, with each test scan registered to the corresponding Artec Eva reference model. The same ICP parameters were applied to all datasets. The algorithm was configured to use a fixed maximum of 200 iterations and a final overlap of 70%, with normals ignored. No additional explicit outlier-rejection procedure was applied beyond the specified overlap setting. Registration was restricted to rigid transformations (translation and rotation), and scaling was explicitly disabled to preserve true dimensional differences between the scanning systems. Following registration, the resulting transformation was applied to the entire cropped facial surface.

Geometric agreement between each test scanner and the Artec Eva reference model was subsequently quantified using Cloud-to-Mesh (C2M) distance analysis. Outcome measures included root mean square error (RMSE), mean and median signed deviation, within-scan standard deviation, 95th percentile deviation, maximum positive and negative deviations, and the proportion of surface points located within predefined tolerance thresholds of ±0.5 mm and ±1.0 mm.

### 2.6. Landmark-Based Agreement Analysis

Anthropometric measurements were obtained from all reconstructed facial models. Inter-landmark distances acquired from each test scanner were compared with corresponding measurements derived from the Artec Eva reference scan. Agreement with the Artec Eva reference was assessed using the mean signed difference (bias), mean absolute error (MAE), and 95% confidence intervals (95% CI).

### 2.7. Statistical Analysis

Statistical analyses were performed using IBM SPSS Statistics, version 31.0.0 (IBM Corp., Armonk, NY, USA). Agreement between scanning systems and the reference scanner was evaluated using Bland–Altman analysis, intraclass correlation coefficients (ICC; two-way mixed-effects model, absolute agreement), and Wilcoxon signed-rank tests. Normality of the data distribution was assessed using the Shapiro–Wilk test. Because normality assumptions were violated for several outcome measures, differences between scanners were analysed using the Friedman test followed by Bonferroni-corrected pairwise post-hoc comparisons. Statistical significance was defined as *p* < 0.05. Scanner-specific descriptive analyses and comparisons with the Artec Eva reference were performed using all available datasets (*n* = 28 for MIRACO and CR-Scan and *n* = 22 for Kiri Engine). Comparisons among all three test scanners using the Friedman test were restricted to the 22 participants with complete datasets for all three test systems.

**Figure 1:**
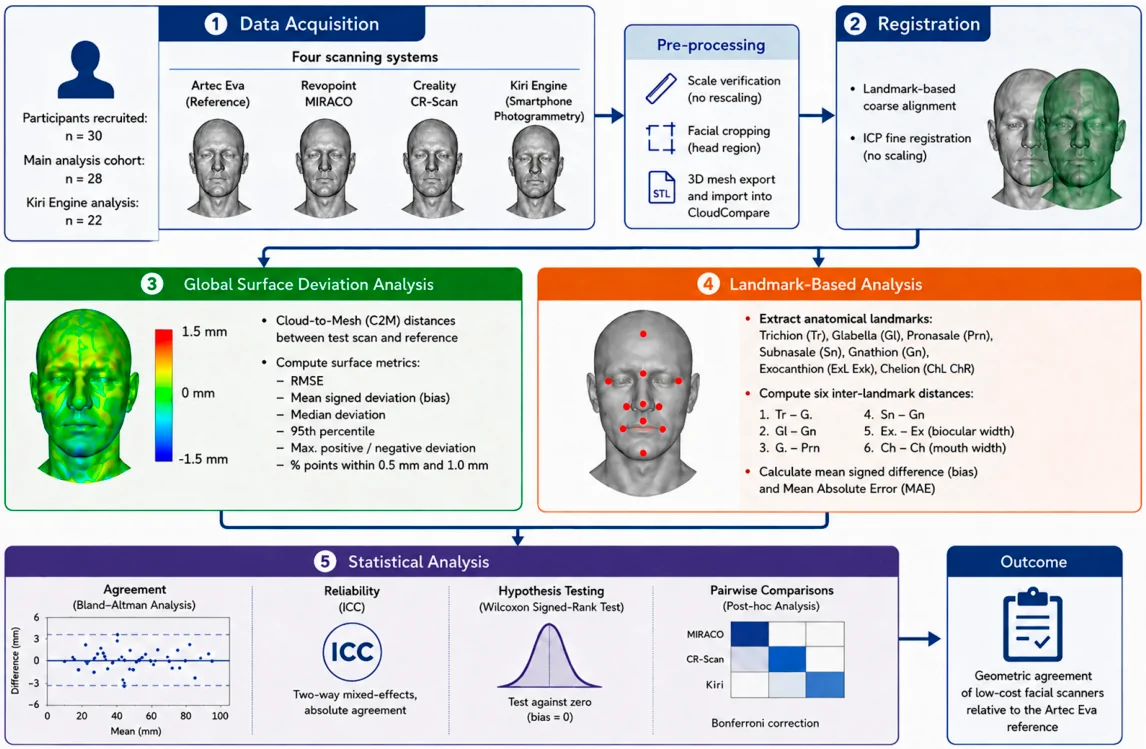
Overview of the study workflow and validation pipeline.

The primary outcome measure was global geometric agreement with the Artec Eva reference surface, assessed using Cloud-to-Mesh deviation metrics. Secondary outcomes included inter-landmark measurement agreement and clinically relevant tolerance-band analysis. The overall processing and validation workflow is summarized in Figure 1.

## 3. Results

### 3.1. Participant Characteristics

Thirty volunteers were recruited for the study. Complete reference scans suitable for analysis were obtained from 28 participants. For Kiri Engine, 22 of 28 acquired datasets met the predefined quality criteria, corresponding to a reconstruction success rate of 78.6%. Six datasets (21.4%) were excluded due to reconstruction failure, incomplete facial coverage, failed mesh generation, or severe geometric artefacts.

Participant demographic characteristics are summarized in Table 1. The mean participant age was 23.7 ± 4.1 years (range 19–43 years). Twenty participants (71%) were female and eight (29%) were male. The majority of participants exhibited Fitzpatrick skin phototypes II and III.

### 3.2. Landmark-Based Measurement Agreement

Results of the inter-landmark distance analysis are summarized in Table 2. Agreement between each test scanner and the Artec Eva reference was assessed using six clinically relevant inter-landmark distances. MIRACO demonstrated small positive biases across all evaluated measurements, ranging from +0.38 mm to +1.16 mm, with mean absolute errors between 1.05 mm and 1.50 mm. CR-Scan exhibited the lowest systematic bias, with mean signed differences generally remaining within ±0.5 mm and MAE values ranging from 0.73 mm to 2.17 mm. Kiri Engine showed greater variability and wider confidence intervals, particularly for trichion–glabella and biocular width measurements, where the largest absolute errors were observed (2.75 mm and 2.14 mm, respectively). Despite differences in individual measurements, all systems demonstrated submillimeter mean biases for most inter-landmark distances. Across all scanners, the smallest deviations were observed for the glabella–pronasale distance.

### 3.3. Bland–Altman Agreement Analysis

**Figure 2:**
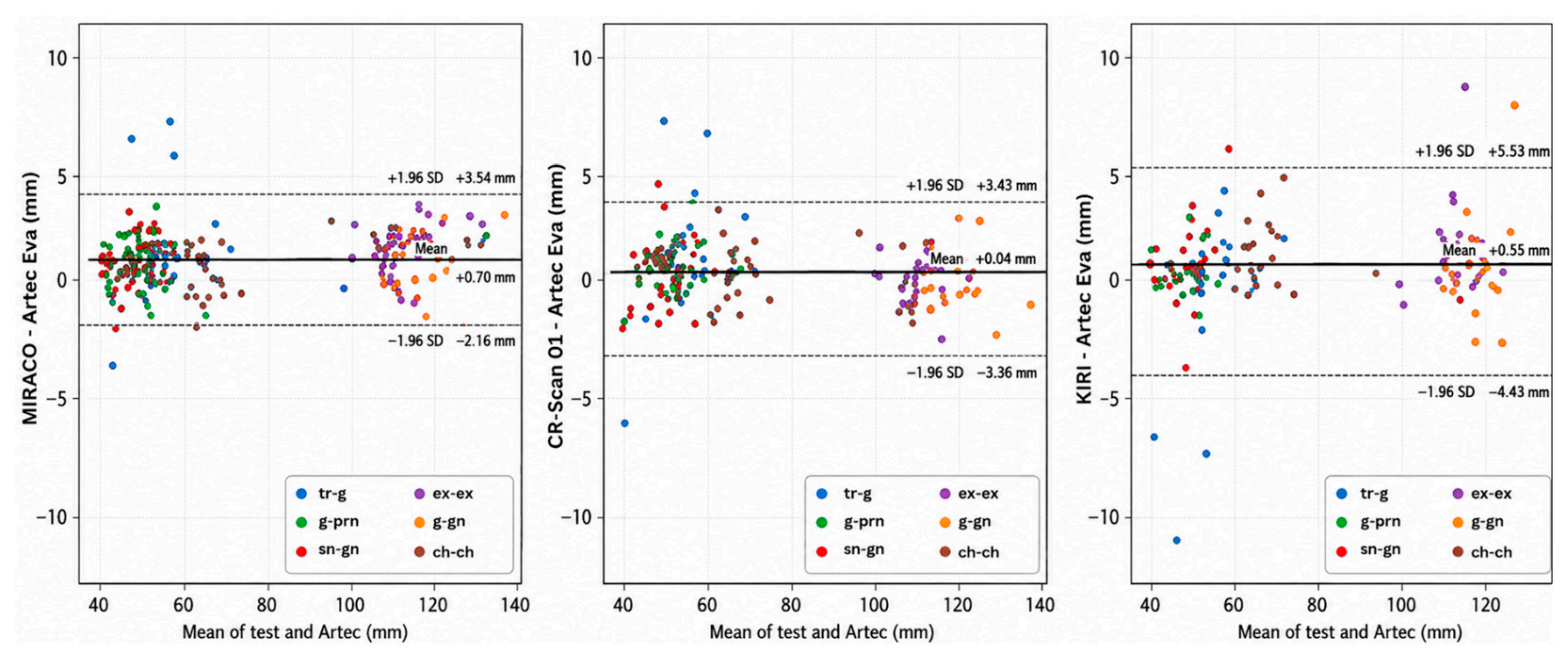
Bland–Altman plots for all test scanners compared with the Artec Eva reference, pooled across six inter-landmark distances.

Results of the Bland–Altman analysis and intraclass correlation assessment are summarized in Table 3, while the agreement patterns and distribution of measurement differences are illustrated in Figure 2. Bland–Altman analysis demonstrated small pooled mean biases but notable differences in the width of the limits of agreement between the evaluated systems. Revopoint MIRACO showed a mean bias of +0.70 mm with limits of agreement ranging from −2.16 mm to +3.54 mm, whereas Creality CR-Scan 01 exhibited the smallest systematic bias (+0.04 mm) among all evaluated systems. In contrast, Kiri Engine demonstrated wider limits of agreement (−4.43 mm to +5.53 mm), indicating greater variability and reduced measurement consistency. Pooled intraclass correlation coefficients across the six inter-landmark distances were high for all scanners (ICC = 0.998–0.999). However, these pooled ICC values reflect relative consistency across measurements and are influenced by the between-subject variability in facial dimensions; therefore, they should not be interpreted as evidence of small absolute measurement differences.

### 3.4. Global Surface Agreement with the Reference

**Figure 3:**
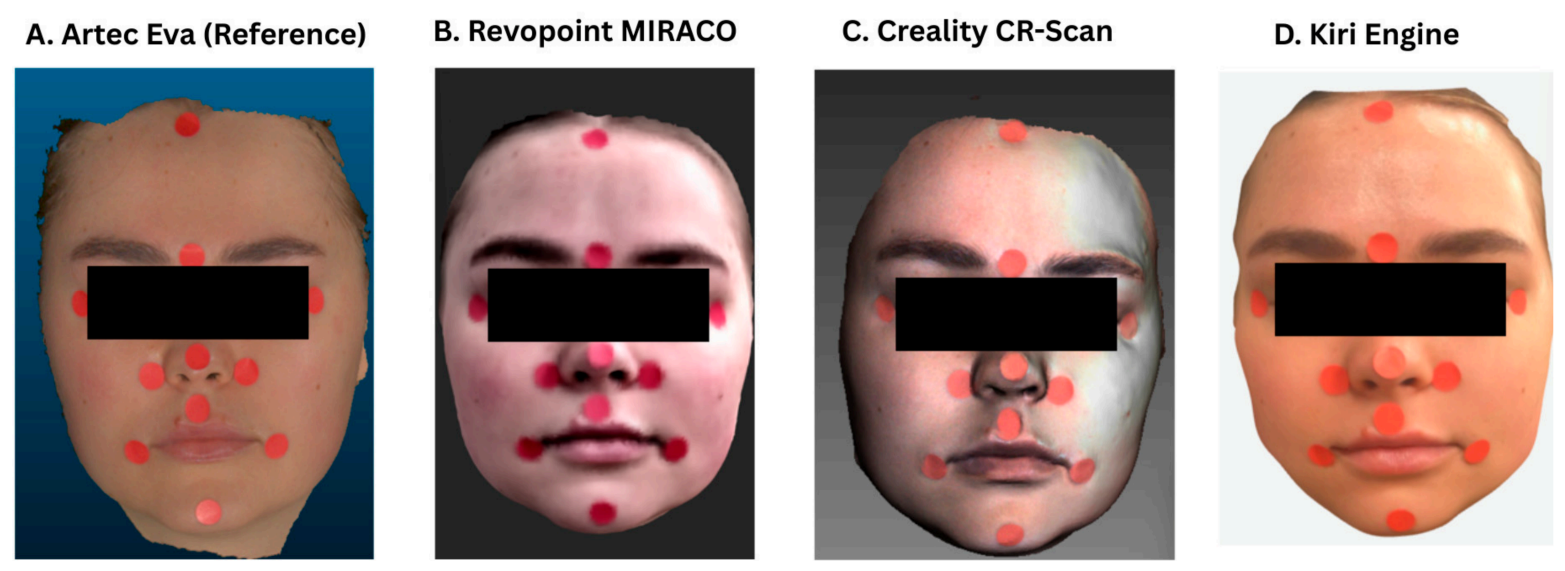
Representative facial reconstructions generated by the evaluated scanning systems: (**A**) Artec Eva reference scan; (**B**) Revopoint MIRACO; (**C**) Creality CR-Scan 01; and (**D**) Kiri Engine smartphone photogrammetry reconstruction. All models are shown in frontal view under identical visualization settings.

Cloud-to-Mesh analysis revealed substantial differences between scanning technologies. Representative facial reconstructions generated by each evaluated scanner are shown in Figure 3A–D.

Global Cloud-to-Mesh deviation metrics relative to the Artec Eva reference are summarized in Table 4. CR-Scan demonstrated the lowest RMSE relative to the Artec Eva reference (0.57 ± 0.17 mm), followed by MIRACO (0.72 ± 0.24 mm). Kiri Engine showed considerably larger relative deviations, with an RMSE of 1.41 ± 0.58 mm.

Mean signed surface bias remained below 0.25 mm for all scanners. The percentage of facial surface points located within 1 mm of the reference model exceeded 90% for both structured-light scanners, whereas Kiri Engine achieved only 68%.

### 3.5. Pairwise Comparisons Between Scanners

Pairwise, post-hoc testing results are presented in Table 5. Pairwise post-hoc testing demonstrated no statistically significant difference between MIRACO and CR-Scan in overall surface deviation relative to the reference (*p* = 0.42). Both structured-light scanners significantly outperformed Kiri Engine (both *p* < 0.001).

### 3.6. Representative Surface Deviation Maps

**Figure 4:**
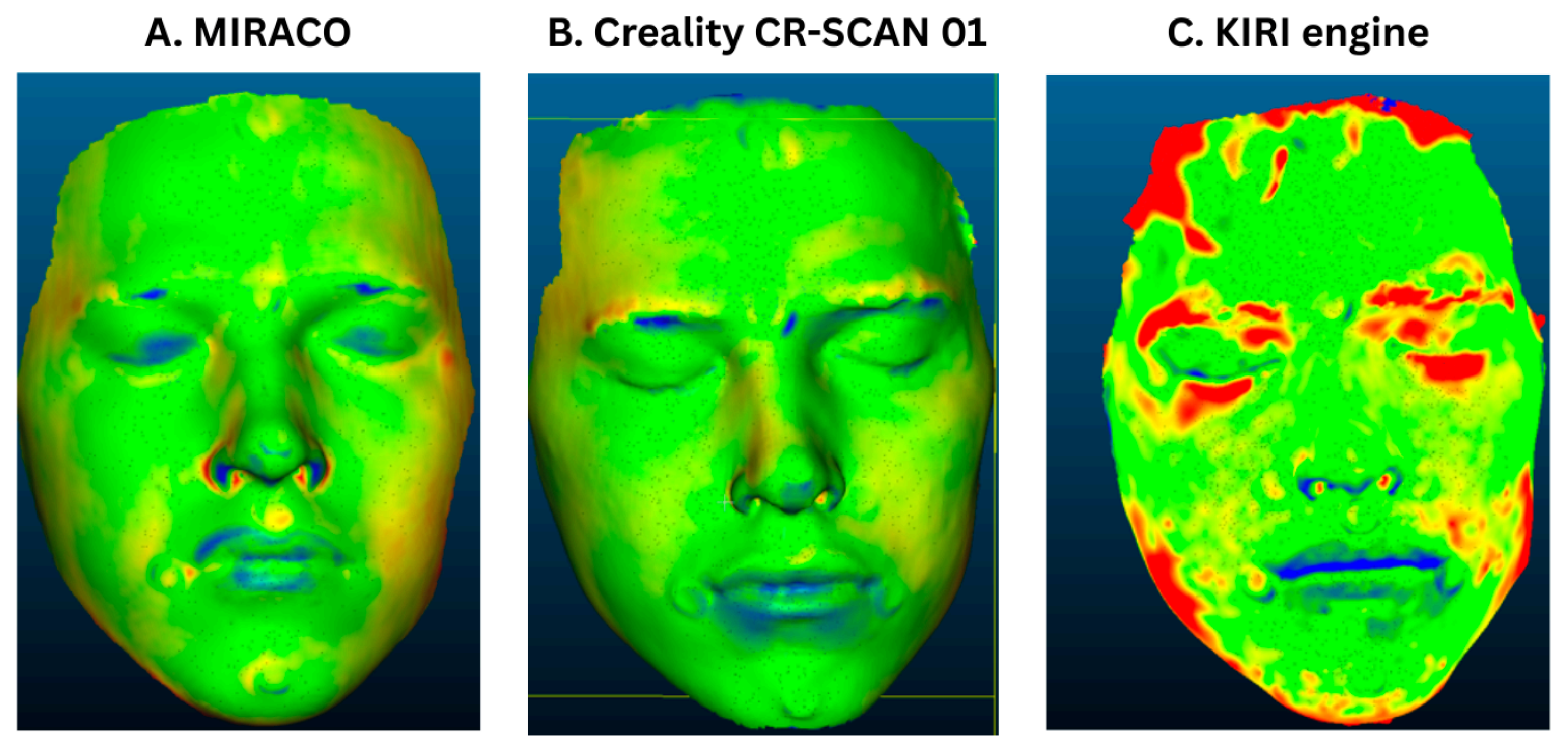
Representative Cloud-to-Mesh deviation maps relative to the Artec Eva reference model: (**A**) Revopoint MIRACO; (**B**) Creality CR-Scan 01; and (**C**) Kiri Engine.

Representative colour-coded deviation maps for a single participant (Subject 25) are presented in Figure 4. All maps were visualized using an identical colour scale saturated at ±1.5 mm to facilitate direct visual comparison between scanners. Subject 25 was selected as a representative example because its global RMSE was closest to the cohort median.

The Revopoint MIRACO reconstruction demonstrated predominantly uniform agreement with the reference model across the central facial region, with only small localized negative deviations observed at the philtrum, alar margins, and chin. A similar pattern was observed for the Creality CR-Scan 01, which exhibited comparable localized deviations around the perioral region and chin, together with a slight positive deviation along the nasal dorsum.

In contrast, the Kiri Engine reconstruction displayed substantially larger and more heterogeneous deviations. Extensive regions of positive deviation exceeding +1.5 mm were observed in the temporal, lateral forehead, and periorbital regions, whereas agreement with the reference surface remained highest around the central nasal and perioral areas.

## 4. Discussion

The present study evaluated the geometric agreement of three low-cost facial scanning systems with the professional structured-light scanner Artec Eva used as a comparative reference. Overall, the two consumer-grade structured-light systems demonstrated better geometric agreement with the reference than smartphone-based photogrammetry, supporting further investigation of affordable structured-light technologies for quantitative facial surface acquisition.

These findings are consistent with previous studies reporting favourable performance of portable structured-light scanners. Pellitteri et al. demonstrated that portable structured-light systems may represent viable alternatives to established stereophotogrammetric systems [17]. In a subsequent study comparing the EinScan H2 with the Vectra M3 reference system, high reproducibility and accuracy were also demonstrated [18]. In contrast, smartphone-based facial scanning has generally shown greater geometric deviations. Van Lint et al. reported deviations of approximately 2–4 mm for smartphone-generated facial models compared with a stereophotogrammetric reference system [19]. The performance of smartphone-based solutions may also depend considerably on the acquisition protocol, reconstruction algorithm, and device hardware [20,21].

The clinical relevance of the observed geometric agreement should be interpreted in relation to the intended application rather than according to a universal accuracy threshold. Although MIRACO and CR-Scan showed favourable global surface agreement with the Artec Eva reference, larger deviations occurred for some individual inter-landmark measurements. These systems may therefore be promising for overall facial surface documentation and monitoring of larger morphological changes, whereas applications requiring precise landmark localization or high local dimensional accuracy, such as detailed surgical planning or fabrication of closely fitting patient-specific devices, require further application-specific validation [12,19].

The very high pooled ICC values should also be interpreted together with the Bland–Altman and surface deviation results. ICC reflects relative agreement and is influenced by the variability of the measurements included in the analysis; therefore, a high ICC does not necessarily indicate small absolute differences between methods. Accordingly, Bland–Altman limits of agreement and absolute deviation metrics provide complementary information that is more directly relevant to the magnitude of measurement differences. This explains why Kiri Engine showed a high pooled ICC despite wider limits of agreement and greater surface deviations relative to the reference.

Reconstruction success represents an additional aspect of practical performance. For Kiri Engine, 22 of 28 acquired datasets met the predefined quality criteria, corresponding to a reconstruction success rate of 78.6%. Because quantitative analysis was restricted to successfully reconstructed models, exclusion of unsuccessful or poor-quality reconstructions may have resulted in an overestimation of its practical geometric performance. Nevertheless, cost and accessibility remain important considerations for clinical implementation, particularly because professional facial imaging systems may require substantially greater financial investment. The approximate costs of the evaluated systems are summarized in Table 6, highlighting the substantial price differences between professional, low-cost, and smartphone-based solutions [12].

**Table 6:** Approximate acquisition costs of evaluated systems.

System	Technology	Approximate Cost (€)
Artec Eva	Structured light	10,000–15,000
Revopoint MIRACO	Structured light	1400–1700
Creality CR-Scan 01	Structured light	600–900
Kiri Engine	Smartphone photogrammetry	<100/year *

* Excluding smartphone hardware.

The present findings are also consistent with previous work by our research group comparing low-cost three-dimensional scanning technologies for biomedical applications. Structured-light scanning showed greater surface agreement with the reference model than smartphone-based approaches [22,23]. Although that investigation was performed on a torso phantom rather than human facial anatomy, it similarly indicated superior geometric fidelity of structured-light acquisition.

Several limitations should be considered. All acquisitions were performed by a single experienced operator, which reduced operator-related variability but prevented assessment of inter-operator reproducibility; intra-device repeatability was also not evaluated. Furthermore, the scanning sequence was fixed, with Artec Eva acquired first, followed by CR-Scan, MIRACO, and Kiri Engine. Although participants were instructed to maintain a neutral facial expression and stable head position, small changes in lip or mandibular position, eyelid configuration, or facial muscle tone may have occurred between consecutive acquisitions. Such physiological variability cannot be quantitatively separated from scanner-related differences in the present study and may be particularly relevant when interpreting submillimetre deviations.

The study population consisted exclusively of healthy adults with intact facial anatomy. The findings therefore cannot be directly generalized to patients with post-burn facial deformities, hypertrophic scars, contractures, edema, or tissue loss, which may alter both surface geometry and optical characteristics. Validation in relevant clinical populations is therefore required before applicability to reconstructive surgery, burn care, or other pathology-specific workflows can be established [24,25].

Finally, Artec Eva was used as a professional comparative reference rather than as an error-free representation of true facial geometry. Although its professional-grade structured-light technology and manufacturer-specified three-dimensional accuracy of up to 0.1 mm supported its selection as the reference system, its measurements are themselves subject to uncertainty. Accordingly, the reported RMSE, Cloud-to-Mesh, and landmark-based deviation values represent geometric differences relative to Artec Eva rather than absolute measurement errors of the investigated scanners.

Overall, affordable structured-light scanners demonstrated favourable geometric agreement with the Artec Eva reference when scanning healthy facial anatomy under controlled conditions. Their portability, accessibility, and geometric performance support further investigation for facial surface documentation and monitoring. However, validation in relevant clinical populations and assessment of repeatability and inter-operator reproducibility are required before broader clinical applicability can be established.

## Figures and Tables

**Table 1:** Demographic characteristics of the study cohort.

Characteristic	Value
Initially recruited participants	30
Participants who withdrew	2
Main study cohort	28
Artec Eva datasets analyzed	28
MIRACO datasets analyzed	28
CR-Scan datasets analyzed	28
Kiri Engine analyzable datasets	22
Kiri Engine datasets excluded due to insufficient quality	6
Age (years)	23.7 ± 4.1
Age range	19–43
Female	20 (71%)
Male	8 (29%)
Fitzpatrick II	10
Fitzpatrick III	14
Fitzpatrick IV	4

**Table 2:** Results of the inter-landmark distance analysis.

Distance	MIRACO Δ [95% CI]	MIRACO MAE	CR-Scan Δ [95% CI]	CR-Scan MAE	Kiri Δ [95% CI]	Kiri MAE
Trichion–Glabella	+0.92 [+0.05, +1.78]	1.50	−0.03 [−1.35, +1.29]	2.17	−1.30 [−3.15, +0.54]	2.75
Glabella–Gnathion	+0.67 [+0.18, +1.16]	1.24	−0.08 [−0.70, +0.55]	1.20	+0.59 [−0.53, +1.71]	1.71
Glabella–Pronasale	+0.38 [−0.08, +0.84]	1.05	+0.12 [−0.23, +0.47]	0.73	+0.41 [−0.09, +0.90]	0.85
Subnasale–Gnathion	+0.69 [+0.22, +1.15]	1.12	+0.20 [−0.47, +0.87]	1.43	+0.75 [+0.03, +1.46]	1.20
Biocular Width (Ex–Ex)	+1.16 [+0.74, +1.57]	1.41	−0.32 [−0.89, +0.24]	0.90	+1.18 [−0.11, +2.48]	2.14
Mouth Width (Ch–Ch)	+0.40 [−0.10, +0.90]	1.20	−0.43 [−1.23, +0.37]	1.44	+0.72 [−0.28, +1.71]	1.72

**Table 3:** Bland–Altman agreement and ICC.

Scanner	Bias (mm)	95% LoA (mm)	ICC (95% CI)
MIRACO	+0.70	−2.16 to +3.54	0.999 [0.999, 1.000]
CR-Scan	+0.04	−3.36 to +3.43	0.999 [0.998, 1.000]
Kiri Engine	+0.55	−4.43 to +5.53	0.998 [0.997, 0.999]

**Table 4:** Global Cloud-to-Mesh deviation metrics relative to the Artec Eva reference.

Metric	MIRACO	CR-Scan	Kiri
RMSE (mm)	0.72 ± 0.24	0.57 ± 0.17	1.41 ± 0.58
Mean bias (mm)	+0.07 ± 0.07	0.00 ± 0.09	+0.21 ± 0.42
% points ≤ 1 mm	91.00 ± 8.00	93.00 ± 7.00	68.00 ± 16.00
% points ≤ 0.5 mm	74.00 ± 12.00	78.00 ± 13.00	45.00 ± 15.00

**Table 5:** Pairwise post-hoc comparisons of global median absolute Cloud-to-Mesh deviation relative to the Artec Eva reference.

Comparison	*p*-Value
MIRACO vs. CR-Scan	0.42
MIRACO vs. Kiri	<0.001
CR-Scan vs. Kiri	<0.001

## Data Availability

The data presented in this study are available on reasonable request from the corresponding author. The data are not publicly available due to privacy and ethical restrictions associated with human participant data and potentially identifiable 3D facial scans.

## References

[1] Chang J.B., Small K., Choi M., Karp N.S. (2015). Three-Dimensional Surface Imaging in Plastic Surgery: Foundation, Practical Applications, and Beyond. Plast. Reconstr. Surg..

[2] Li Y., Yang X., Li D. (2016). The Application of Three-Dimensional Surface Imaging System in Plastic and Reconstructive Surgery. Ann. Plast. Surg..

[3] Kittrick A.M., Brown J. (2025). 110 Implementation of 3D Technology for Management of Facial Scars Across the Spectrum of Care. J. Burn Care Res..

[4] Nguyen C., Nicolai E.S.J., He J.J., Roshchupkin G.V., Corten E.M.L. (2022). 3D Surface Imaging Technology for Objective Automated Assessment of Facial Interventions: A Systematic Review. J. Plast. Reconstr. Aesthet. Surg..

[5] Petrides G., Clark J.R., Low H., Lovell N., Eviston T.J. (2021). Three-Dimensional Scanners for Soft-Tissue Facial Assessment in Clinical Practice. J. Plast. Reconstr. Aesthet. Surg..

[6] Zhao Y.J., Xiong Y.X., Wang Y. (2017). Three-Dimensional Accuracy of Facial Scan for Facial Deformities in Clinics: A New Evaluation Method for Facial Scanner Accuracy. PLoS ONE.

[7] Antonacci D., Caponio V.C.A., Troiano G., Pompeo M.G., Gianfreda F., Canullo L. (2023). Facial Scanning Technologies in the Era of Digital Workflow: A Systematic Review and Network Meta-Analysis. J. Prosthodont. Res..

[8] Schipper J.A.M., Merema B.J., Hollander M.H.J., Spijkervet F.K.L., Dijkstra P.U., Jansma J., Schepers R.H., Kraeima J. (2024). Reliability and Validity of Handheld Structured Light Scanners and a Static Stereophotogrammetry System in Facial Three-Dimensional Surface Imaging. Sci. Rep..

[9] Singh P., Bornstein M.M., Hsung R.T., Ajmera D.H., Leung Y.Y., Gu M. (2024). Frontiers in Three-Dimensional Surface Imaging Systems for 3D Face Acquisition in Craniofacial Research and Practice: An Updated Literature Review. Diagnostics.

[10] Cascos R., Ortiz Del Amo L., Álvarez-Guzmán F., Antonaya-Martín J.L., Celemín-Viñuela A., Gómez-Costa D., Zafra-Vallejo M., Agustín-Panadero R., Gómez-Polo M. (2023). Accuracy between 2D Photography and Dual-Structured Light 3D Facial Scanner for Facial Anthropometry: A Clinical Study. J. Clin. Med..

[11] D’Ettorre G., Farronato M., Candida E., Quinzi V., Grippaudo C. (2022). A Comparison between Stereophotogrammetry and Smartphone Structured Light Technology for Three-Dimensional Face Scanning. Angle Orthod..

[12] Mai H.N., Lee D.H. (2020). Accuracy of Mobile Device-Compatible 3D Scanners for Facial Digitization: Systematic Review and Meta-Analysis. J. Med. Internet Res..

[13] Nightingale R.C., Ross M.T., Cruz R.L.J., Allenby M.C., Powell S.K., Woodruff M.A. (2021). Frugal 3D Scanning Using Smartphones Provides an Accessible Framework for Capturing the External Ear. J. Plast. Reconstr. Aesthet. Surg..

[14] Jindanil T., Xu L., Fontenele R.C., Perula M.C.L., Jacobs R. (2024). Smartphone Applications for Facial Scanning: A Technical and Scoping Review. Orthod. Craniofac. Res..

[15] König J., Kelemen K., Czumbel L.M., Szabó B., Varga G., Borbély J., Németh O., Hegyi P., Hermann P. (2024). Current Status of Optical Scanning in Facial Prosthetics: A Systematic Review and Meta-Analysis. J. Prosthodont. Res..

[16] Hou X., Xu X., Zhao M., Kong J., Wang M., Lee E.S., Jia Q., Jiang H.B. (2022). An Overview of Three-Dimensional Imaging Devices in Dentistry. J. Esthet. Restor. Dent..

[17] Pellitteri F., Scisciola F., Cremonini F., Baciliero M., Lombardo L. (2023). Accuracy of 3D Facial Scans: A Comparison of Three Different Scanning Systems in an In Vivo Study. Prog. Orthod..

[18] Pellitteri F., Calza M., Baldi G., De Maio M., Lombardo L. (2025). Reproducibility and Accuracy of Two Facial Scanners: A 3D In Vivo Study. Appl. Sci..

[19] Van Lint L., Christiaens L., Stroo V., Bila M., Willaert R., Sun Y., Van Dessel J. (2023). Accuracy Comparison of 3D Face Scans Obtained by Portable Stereophotogrammetry and Smartphone Applications. J. Med. Biol. Eng..

[20] Tangthaweesuk N., Raocharernporn S. (2025). The Accuracy of Three-Dimensional Facial Scans Obtained from Three Different 3D Scanners. PLoS ONE.

[21] Kamrani P., Munavalli G. (2024). Update on Three-Dimensional Imaging Technologies and Their Impact and Use in Cosmetic Surgery. Adv. Cosmet. Surg..

[22] Ondrejová B., Štefanovič B., Bednarčíková L., Tóth T., Živčák J. (2024). 3D Scanning of the Maxillofacial Area for Individual Mask Production. Proceedings of Novus Scientia 2024.

[23] Štefanovič B., Ondrejová B., Bednarčíková L., Tóth T., Živčák J. Comparison of Optical Handheld 3D Scanners Suitable for Prosthetic and Orthotic Applications. Proceedings of the 13th International Conference and Exhibition on 3D Body Scanning and Processing Technologies (3DBODY.TECH 2022).

[24] Dudová K., Ondrejová B., Demčák T., Michalíková M., Bednarčíková L., Živčák J., Lengyel P., Eliáš E. (2026). Three-Dimensional Visualisation of Burn Wounds: Concordance of Artec Eva and Revopoint Miraco with Clinical Photography—A Case Series. Eur. Burn J..

[25] Ondrejová B., Dudová K., Michalíková M., Bednarčíková L., Živčák J., Demčák T., Lengyel P. (2026). Objective Longitudinal Monitoring of Burn Wound Area Using 3D Surface Scanning: A Pilot Study. Eur. Burn J..

